# EXAMINING AGING AMONG GUANAJA, HONDURAS ISLANDERS THROUGH THE UTILIZATION OF SOCIAL EXCLUSION FRAMEWORKS

**DOI:** 10.1093/geroni/igad104.3314

**Published:** 2023-12-21

**Authors:** Lee Ann Ferguson

**Affiliations:** UMBC Erickson School of Aging Studies, Baltimore, Maryland, United States

## Abstract

Guanaja is an island municipality of Honduras located approximately 70 miles from its parent island . Of the country’s three main island municipalities, it is the second largest. The central municipality of Guanaja is located less than half of a mile from the coast of Guanaja and is a 100-acre floating island city built over two smaller cays which consists of densely compacted, stilt-built homes and houses approximately 3,000 people. It is estimated that 6.6% of the total population are over the age of 65. The current body of both spatial and social exclusion research considers environmental conditions may act in ways which may restrict or deny access or choices for aging inhabitants. Due to its remote geographic location, the island of Guanaja reflects key risk factor for age-related social and spatial exclusion. Framed by two social exclusion frameworks, this study, which was the cornerstone of this author’s dissertation, presents the reality of island aging among Guanajan residents as relayed by them during this focused ethnographic study. The results of this work will describe the influences on Guanajan aging from factors including geographic environment and location, neighborhood and community, civic engagement and participation, social relationships, material and financial resources, socio-cultural practices, and governmental policies and practices.

